# Regulatory T Cells for Stroke Recovery: A Promising Immune Therapeutic Strategy

**DOI:** 10.1111/cns.70248

**Published:** 2025-01-29

**Authors:** Ning Li, Hujun Wang, Changbin Hu, Shuyan Qie, Zongjian Liu

**Affiliations:** ^1^ Department of Rehabilitation, Beijing Rehabilitation Hospital Capital Medical University Beijing China; ^2^ Department of Research, Beijing Rehabilitation Hospital Capital Medical University Beijing China

**Keywords:** ischemic stroke, neuroinflammation, stroke recovery, Treg cells

## Abstract

**Background:**

Stroke remains a leading cause of mortality and disability among adults. Given the restricted therapeutic window for intravascular interventions and neuroprotection during the acute phase, there has been a growing focus on tissue repair and functional recovery in the subacute and chronic phases after stroke. The pro‐inflammatory microglial polarization occurs in subacute and chronic phases after stroke and may represent therapeutic targets for stroke recovery. CD4^+^ regulatory T cells (Tregs), a subtype of T cells with immunosuppressive effects, have been shown to be important in stroke. Tregs infiltrate into the brain primarily during the subacute and chronic phases following a stroke. Infiltrating Tregs play a critical role in mitigating pro‐inflammatory microglial responses, modulating the immune microenvironment, and promoting the functional restoration of the damaged brain following a stroke.

**Methods:**

A systematic literature search was conducted in PubMed, Scopus, and Web of Science and then conduct a comprehensive analysis of the searched literature.

**Results:**

This review provides a comprehensive summary of recent preclinical research advances on the role of Tregs in stroke, with a particular focus on their reparative functions during the subacute and chronic phases. It discusses changes in peripheral and brain infiltrating Tregs post‐stroke, their functions and underlying mechanisms, and therapeutic strategies involving Tregs. Additionally, this review explores the potential and challenges associated with the clinical application of Tregs in ischemic stroke.

**Conclusion:**

Treg cell‐related therapy represents a promising immune‐therapeutic strategy for stroke recovery. However, there are several critical issues that must be resolved before its advancement to clinical application.

## Graphical abstract

Both adoptive transfer of the expanded Tregs and increasing Treg numbers via agents that can stimulate endogenous Treg proliferation are therapeutic strategies for enhancing Treg levels in stroke. Tregs play a crucial role in neuroprotection and functional recovery after stroke by restoring the integrity of the blood–brain barrier, suppressing the immune response, restoring myelin integrity, and suppressing astrogliosis and neurotoxicity.

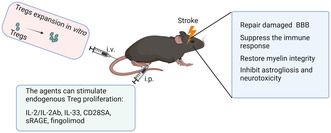

## 1 Introduction

Stroke, a condition associated with high mortality and disability, has seen a significant increase in global incidence and prevalence over the past few decades. In particular, the incidence of stroke in young patients has increased [1]. Ischemic stroke (IS) is the most common type of stroke. The primary therapeutic approach for IS focuses on achieving timely recanalization and restoring blood supply within the critical therapeutic window, primarily through intravenous thrombolysis and endovascular thrombectomy. In the acute phase of IS, a cascade of pathological processes—including neurotoxicity, oxidative stress, and inflammatory responses—triggers various modes of cell death, such as apoptosis, necroptosis, ferroptosis, pyroptosis, and parthanatos. These processes collectively contribute to the high mortality and disability associated with IS [2]. Accordingly, the inhibition of neuronal death in the region surrounding the infarct core to protect the damaged brain is also important during the acute phase. However, owing to time constraints in the acute phase, recovery strategies for the subacute and chronic phases after stroke are receiving increasing attention. During these phases, the primary focus is on neurofunctional recovery. This recovery is underpinned by neuroplasticity, which encompasses processes such as synaptic plasticity, neurogenesis, angiogenesis, and axonal regeneration, all of which are essential for functional restoration after IS.

The inflammatory response is an important pathological process after stroke, and a variety of immune cells are involved in this process, including resident immune cells in the brain such as microglia and astrocytes, as well as infiltrating peripheral immune cells such as neutrophils, monocytes, T cells, and B cells [3]. In the acute phase, the activation of some immune cells can cause ischemic damage while also having a certain protective effect. However, excessive inflammatory responses can lead to secondary injuries after stroke [4]. CD4^+^ regulatory T cells (Tregs), as a kind of immunomodulatory cells, can interact with multiple immune cells to play roles in alleviating the inflammatory response after stroke and protecting the blood–brain barrier [5, 6].

Moreover, Tregs contribute to long‐term neuroprotection by directing microglia toward an anti‐inflammatory phenotype and facilitating tissue repair during stroke recovery [6]. Following IS, Tregs infiltrate the ischemic core and peri‐infarct areas in the mouse brain. Different from other peripheral immune cells, the number of recruited Tregs increases significantly between days 7 and 14 post‐stroke and remains high until day 60 [7, 8]. This timeframe coincides with the transition between two distinct polarized subtypes of activated microglia and aligns with the period of neuroplasticity changes following stroke [9]. Hu et al. demonstrated that selective depletion of Tregs reduced myelin coverage 21 days post‐IS. Adoptive transfer of wild‐type (WT) Tregs to Treg‐depleted mice restored myelin integrity. Conversely, no significant difference was observed between Treg depletion and nondepletion at 5 days post‐IS, suggesting that the beneficial effects of Tregs might be partially attributed to promoting late‐phase tissue repair rather than early‐phase neuroprotection [8].

Previous studies have emphasized the neuroprotective role of Tregs during the acute phase after IS. Given the temporal changes in Treg levels after IS, these cells are likely to play a significant role in facilitating recovery during the rehabilitation phase. In this review, we focus on exploring the potential beneficial effects of Tregs in IS rehabilitation and summarize the preclinical research advances on the therapeutic effects of Tregs after IS.

## 2 Basic Characterization of Tregs

Tregs were first identified 50 years ago as a subpopulation of suppressor T cells characterized by the expression of CD4, CD25, and the transcription factor Forkhead box protein P3 (Foxp3). These cells play a crucial role in maintaining immune homeostasis and preventing autoimmune diseases [10]. FoxP3 is essential for regulating the differentiation and development of Tregs, as well as for maintaining their immunosuppressive functions. Additionally, Tregs express glucocorticoid‐induced tumor necrosis factor receptor (GITR), CD103, cytotoxic T‐lymphocyte‐associated protein 4 (CTLA4), Helios, signal transducer and activator of transcription 5 (STAT‐5), and aryl hydrocarbon receptor (AhR), contributing to Treg cell phenotype [11, 12].

Tregs can be broadly categorized into two subsets: thymus‐derived Tregs (tTregs) and peripheral‐derived Tregs (pTregs). tTregs, also known as naturally occurring Tregs (nTregs), constitute the majority of the total Treg population. Their differentiation is linked to the high‐affinity interaction of autopeptides with major histocompatibility complex class II (MHC II) molecules in the thymus [13]. Transcription factors Helios, Neuropilin (Nrp), and glycoprotein A33 (GPA33) are the specific markers for recognizing tTregs [14, 15, 16]. pTregs originate from naive CD4^+^ T cell in the periphery, and their differentiation occurs upon antigen stimulation with certain related cytokines, including interleukin 2 (IL‐2) and transforming growth factor‐β (TGF‐β) [17].

Tregs are located in lymphoid and various nonlymphoid tissues, such as the skeletal muscle, skin, lung, colon, adipose tissue, intestine, and brain, and so on [18, 19, 20]. Tregs in local areas can express genes with biological features common to Tregs as well as those specific to resident tissues [5]. Brain‐resident Tregs express common Treg markers such as CTLA‐4, Helios, GITR, and programmed death‐1 (PD‐1). In addition, they uniquely express genes associated with the nervous system, including serotonin receptor type 7 (Htr7), arginine vasopressin receptor (Avpr1a), preproenkephalin (Penk), and neuropeptide Y (Npy) [7, 21]. In addition to their immunosuppressive functions, brain‐resident Tregs play roles in promoting tissue repair and maintaining tissue homeostasis [18, 22]. The transcriptional and protein expression levels of FoxP3 are crucial for ensuring the stability of FoxP3^+^ Tregs [23]. Therefore, transcription factors that affect FoxP3 transcription, such as cAMP response element binding (CREB), and CD98 and CD147 expressed on Tregs—which affect the stability of the FoxP3 protein—can also influence the immunosuppressive functions of Tregs [10, 24].

## 3 Changes of Tregs in IS

Under normal physiological conditions, Tregs maintain a homeostatic state and comprise only 5%–10% of circulating T cells [25]. However, in nervous system pathologies, a reduction in Treg cell numbers or dysfunction of Tregs is often observed during the early phases of these conditions [26]. Diseases directly associated with inflammatory disorders, such as multiple sclerosis (MS), Guillain–Barré syndrome (GBS), and chronic inflammatory demyelinating polyradiculoneuropathy (CIDP), are known to reduce circulating Treg numbers [27, 28, 29]. Similarly, neurodegenerative diseases such as Parkinson's disease (PD) and Alzheimer's disease (AD) also show reduced Treg levels in peripheral blood during the early stages of disease progression compared to healthy controls [30, 31, 32]. Moreover, Treg depletion has been found to correlate with increased disease severity in these conditions.

In contrast to slowly progressive diseases, IS is a sudden event characterized by ischemic and hypoxic injury to brain tissue. Following the onset of stroke, both the absolute number and functional capacity of peripheral Tregs are significantly reduced in clinical patients [6, 33, 34]. However, the proportion of Tregs in peripheral blood increases after stroke due to the significant apoptosis of total lymphocytes, with Tregs experiencing a relatively milder reduction compared to other lymphocyte subsets [35, 36, 37]. In IS patients, the number of Tregs in peripheral blood surpasses that of healthy controls by day 7 poststroke and continues to rise until the third week [37, 38]. Additionally, studies have shown that the percentage of CD4^+^ Tregs in acute IS patients correlates with neurofunctional deficits during both the acute and long‐term phases of IS. This suggests a strong association between circulating Treg levels and stroke outcomes [39]. The level of Tregs found in intracranial thrombi during surgical intervention has been shown to inversely correlate with the incidence of hemorrhagic transformation following thrombectomy [40]. However, several studies have indicated that while the number of Tregs is not a reliable predictor of IS outcome, the proportion of specific Treg cell subtypes provides a more accurate prognostic indicator. Tregs expressing Helios, referred to as H^+^ Tregs, constitute a significantly larger proportion of the total Treg population compared to Helios‐negative (H^−^) Tregs. The proportion of H^+^ Tregs among total Tregs at 3 days poststroke has been shown to predict both clinical outcomes and infarct volume at 90 days poststroke [41]. Moreover, the frequency of naïve Tregs (nTregs) and the nTreg‐to‐memory Treg (mTreg) ratio within the first week after IS are negatively correlated with unfavorable outcomes at 90 days poststroke [42]. These findings suggest that Tregs could serve as valuable biomarkers for predicting stroke outcomes and guiding subsequent therapeutic strategies.

The changes in circulating Tregs observed in IS animal models are consistent with those seen in IS patients. In middle cerebral artery occlusion (MCAO) models, Treg levels decrease significantly 1 day after ischemic injury but subsequently rise, returning to normal levels by day 3 [5]. Seven days after MCAO, the proportion of Tregs in the blood continues to rise [5]. In addition, the levels of Tregs in the spleen fluctuate with changes in the spleen size. Four days poststroke, the number of Tregs in the spleen increases, coinciding with significant splenic atroph [43]. The level of Tregs in the spleen of MCAO mice is also higher than that in the sham group [44].

**FIGURE 1:**
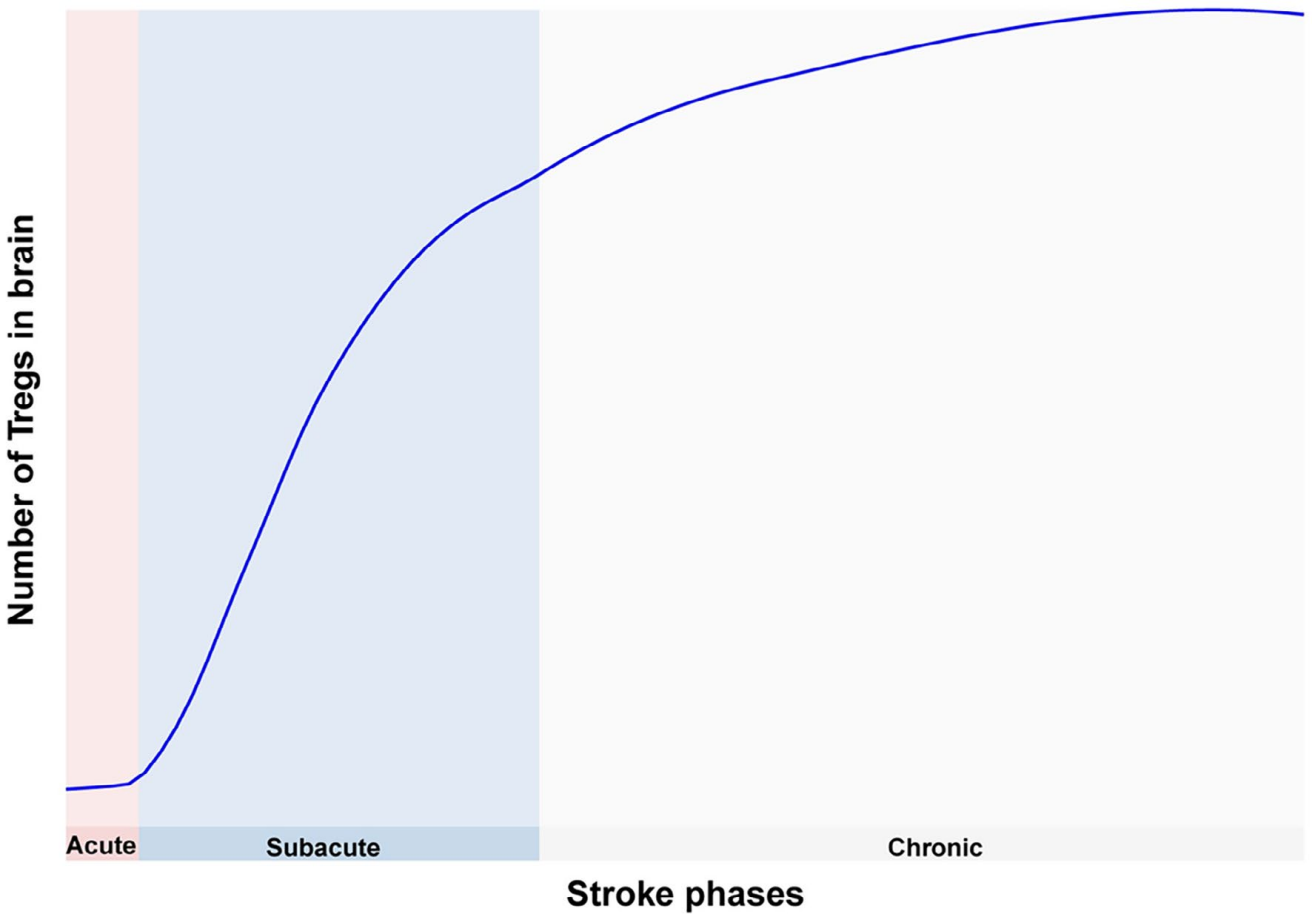
The changes of infiltrating Tregs in brain across stroke phases.

The brain, being the most severely affected organ in IS, exhibits changes in Tregs that differ from those observed in peripheral tissues. Under normal physiological conditions, the number of Tregs in the brain is minimal. Following IS onset, only a small number of Tregs are found in the ischemic area, with their percentage among total CD4^+^ T cells remaining below 5% during the first week after MCAO [45]. The levels of Tregs in both the ischemic core and peri‐infarct areas increase significantly between days 7 and 14 following MCAO and remain elevated until day 60 [7, 8, 22] (Figure 1). However, in nonreperfusion models, Tregs increase to approximately 20% in the ischemic hemisphere by 3 days after MCAO [46]. The timing of significant Treg infiltration into the brain may vary depending on whether the animal model involves reperfusion or nonreperfusion following ischemia.

The increase in Tregs within the ischemic brain following IS depends on the chemokine‐mediated recruitment of peripheral Tregs to the ischemic core and peri‐infarct areas. C‐C chemokine receptor type 5 (CCR5) is prominently expressed on circulating Tregs following MCAO. Concurrently, C‐C motif chemokine ligand 5 (CCL5), a CCR5 ligand, is highly expressed in the endothelium. This expression facilitates Tregs docking with the ischemic endothelium and interacting with neutrophils and macrophages. These interactions ultimately contribute to protecting against early blood–brain barrier (BBB) disruption following stroke [47]. Several chemokines facilitate Treg recruitment during the chronic phase of IS. The expression of CCR8 and CCR6 on Tregs increases between days 7 and 14 post‐IS. Concurrently, CCL1, a CCR8 ligand, and CCL20, a CCR6 ligand, are highly expressed in astrocytes and oligodendrocytes, respectively, within the ischemic brain [7]. This suggests that these chemokines may trigger Tregs migration in the subacute and chronic phases after IS. Additionally, CCR4 is highly expressed on Tregs after oxygen–glucose deprivation (OGD) and facilitates enhanced Treg mobility for migration following MCAO [48].

## 4 Effect and Mechanism of Tregs After IS

### 4.1 The Responses of Peripheral Tregs After IS

The effects of Tregs vary across different tissues and phases following IS, corresponding to their dynamic levels. Immediately after stroke onset, Tregs are scarce in the brain, and their numbers in the blood sharply decrease. The functional significance of Tregs during this early phase of IS has been demonstrated in studies that manipulate Treg levels prior to inducing injury in the MCAO model. Depletion of Tregs exacerbates brain damage and worsens functional outcomes, whereas enhancing Treg levels significantly reduces infarct volumes and alleviates behavioral deficits [46, 49]. However, several reports have shown that reducing Tregs prior to MCAO results in smaller infarct sizes and improved neurological function [50, 51]. The variability in these findings may stem from differences in the methods used to reduce Tregs and the timing of their assessment following MCAO. While the reduction of Tregs prior to MCAO has produced inconsistent outcomes, the adoptive transfer of allogeneic Tregs immediately after MCAO has consistently demonstrated a reduction in brain injury and improved neurological function [52, 53].

**FIGURE 2:**
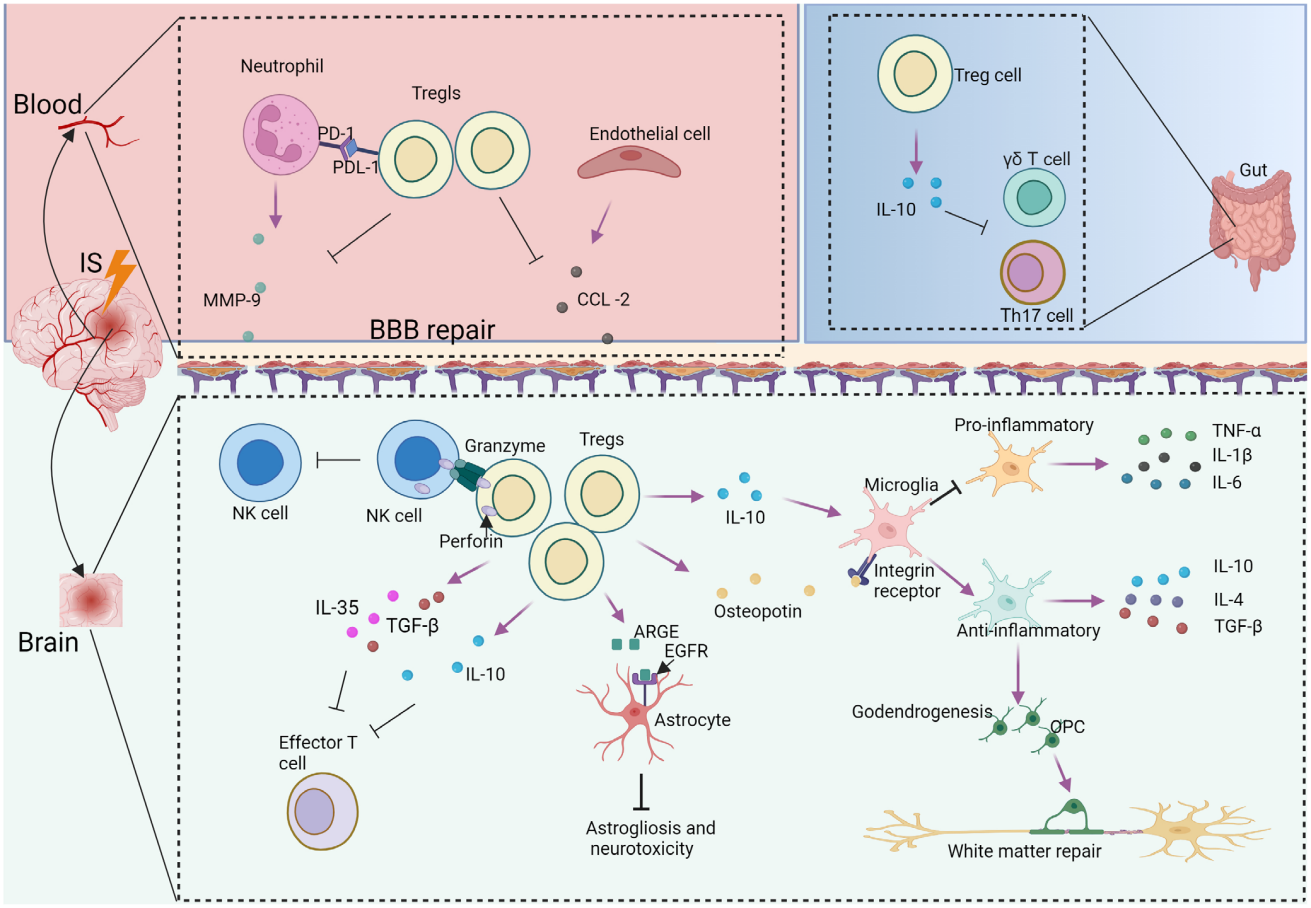
The mechanisms of Tregs protecting against ischemic stroke. Tregs restore the integrity of BBB by inhibiting neutrophil‐derived MMP‐9 and endothelial cell‐derived CCL‐2. In the gut, Tregs suppress γδ T and Th17 cells via IL‐10. In addition, Tregs suppress the immune response through the perforin/granzyme pathway and secreting cytokines such as IL‐10, TGF‐β, and IL‐35. Through IL‐10, Tregs induce a shift in microglial gene expression toward a profile related to pro‐regenerative functions, similar to the osteopontin/integrin receptor, which promotes oligodendrogenesis. Tregs suppress astrogliosis and neurotoxicity in astrocytes through EGFR/AREG.

Before significant infiltration of Tregs into the ischemic brain during the early phase of IS, their primary mechanism of action in IS is the restoration of BBB integrity. Matrix metallopeptidase 9 (MMP‐9), which is elevated in the blood of IS patients within 24 h post‐stroke, contributes to increased BBB permeability and disruption [54]. Tregs inhibit neutrophil‐derived MMP‐9 through enhanced programmed death ligand 1 (PDL‐1)/ PD‐1 interaction [55], similar to their inhibition of endothelial cell‐derived CCL‐2 [33], thereby contributing to the protection of BBB integrity. Moreover, Tregs play a critical role in suppressing systemic inflammatory responses. IS triggers not only localized inflammation in the ischemic brain but also peripheral inflammation, which is strongly associated with rapid neurological decline and poorer functional outcomes. Tregs suppress MCAO‐induced increases in inflammatory cytokines such as interleukin‐6 (IL‐6) and tumor necrosis factor‐α (TNF‐α) in the blood [56]. Additionally, they inhibit the activation of effector T cells and reduce the production of their key effector cytokine, interferon gamma (IFN‐γ) [49]. Another pathway through which peripheral Tregs affect poststroke brain injury is the brain–gut axis. There is a bidirectional regulatory pathway between the brain and the gut involving neural pathways, the hypothalamic–pituitary–adrenal axis, the immune system, neuropeptides, neurotransmitters, and microbial metabolites [57]. After the onset of stroke, gut microbiota dysbiosis affects the inflammatory response and exacerbates brain injury through the brain–gut axis [58]. Following IS, the gut microbiota contributes to Treg polarization by modulating intestinal dendritic cells [59]. The increased Tregs in gut suppress the differentiation of T helper 17 (Th17) cells and the proliferation of effector IL‐17^+^ γδ T cells through IL‐10. This leads to reduced migration of effector T cells from the intestine to the brain, thereby alleviating neuroinflammation in ischemic brain tissue [59, 60] (Figure 2).

### 4.2 The Protective and Restorative Function of Infiltrating Tregs in Stroke‐Damaged Brain

The number of infiltrating Tregs in the ischemic brain begins to increase significantly around 5–7 days after MCAO, accompanied by a rise in peripheral Treg levels. During this phase, Tregs not only exert neuroprotective effects but also actively promote tissue repair and recovery.

Under normal circumstances, Tregs achieve their neuroprotective and recovery effects after stroke by suppressing the inflammatory response. Tregs modulate immune responses following IS by secreting anti‐inflammatory cytokines, including IL‐10, TGF‐β, and IL‐35. IL‐10 is a neuroprotective cytokine that plays a crucial role in dampening poststroke pro‐inflammatory responses, partly through its effects on antigen‐presenting cells [61]. Supplementary administration of IL‐10 has been shown to reduce elevated levels of pro‐inflammatory cytokines in Treg‐depleted mice. Conversely, the administration of Tregs lacking IL‐10 failed to exert immunosuppressive and neuroprotective effects, suggesting that the anti‐inflammatory properties of Tregs following IS are largely mediated through IL‐10 secretion [46]. Additionally, Treg‐derived TGF‐β and IL‐35 play critical roles in immune suppression. The expression of TGF‐β after stroke has been found to correlate with changes in Treg numbers [62, 63]. Treg‐derived TGF‐β also reduces the cytotoxic activity of CD8^+^ T cells [59]. IL‐35 produced by Tregs plays a crucial role in inducing T‐cell exhaustion and suppressing the activity of effector T cells [6]. Additionally, Tregs can modulate immune responses via cell lysis pathways mediated by perforin and granzyme. Activated Tregs are capable of expressing granzyme and inducing the death of activated CD4^+^ and CD8^+^ T cells, natural killer (NK) cells, cytotoxic T lymphocytes, and other cell types through perforin‐dependent and granzyme‐dependent mechanisms [64]. However, there are currently no reports demonstrating that Tregs suppress pro‐inflammatory responses via perforin and granzyme pathways specifically in the context of IS (Figure 2).

On the other hand, Tregs play a crucial anti‐inflammatory role in the brain following IS, primarily due to the presence of sufficient immune cells within the brain that can interact with Tregs and amplify their immunosuppressive effects during subacute and chronic phases [65]. Previous studies have demonstrated that brain‐infiltrating Tregs exhibit a strong propensity to activate phagocytic cells [8], with microglia—brain‐resident innate immune cells—being the primary target. Following IS, microglia can polarize into two distinct phenotypes: pro‐inflammatory microglia, which contribute to exacerbating brain damage, and anti‐inflammatory microglia, which provide neuroprotective and reparative benefits [66]. Spatiotemporal changes in microglial activation and polarization after IS coincide with poststroke neuroplastic changes [67]. Accumulating evidence indicates that microglial polarization plays a critical role in neuroplasticity‐dependent stroke recovery [68]. Consequently, several interventions have been shown to mitigate microglia‐mediated neuroinflammation by suppressing pro‐inflammatory polarization and enhancing anti‐inflammatory polarization following IS [68, 69, 70]. Adoptive transfer of Tregs promotes a shift in microglial gene expression toward a pro‐regenerative profile, primarily mediated by Treg‐derived IL‐10 [71]. Furthermore, the depletion of microglia diminishes the beneficial effects of transferred Tregs on white matter regeneration following IS. The mechanism by which Tregs influence microglia to promote white matter repair involves Treg‐derived osteopontin. Osteopontin targets microglia via integrin receptors. This interaction supports oligodendrogenesis and facilitates white matter repair following IS [8]. These findings highlight the critical role of Tregs in promoting tissue repair after IS through their interactions with microglia. Tregs not only drive microglia toward an anti‐inflammatory phenotype [72] but are also influenced by anti‐inflammatory microglia, which facilitate Treg differentiation and recruitment into the brain via microglia‐derived cytokines such as IL‐4, IL‐10, and TGF‐β [73]. This bidirectional crosstalk between Tregs and microglia amplifies the reparative and immunosuppressive effects of Tregs within the ischemic brain (Figure 2).

Astrocytes, another essential type of glial cell in the brain, play a pivotal role in maintaining homeostasis and structural integrity within the central nervous system [74]. Post‐stroke, Tregs contribute to functional recovery by influencing astrocytes. Following IS, the number of reactive astrocytes increases in a process known as astrogliosis, which occurs primarily in the peri‐infarct areas. The formation of a glial scar by these reactive astrocytes serves as a protective barrier, isolating the ischemic regions from nonischemic areas. During the acute phase of IS, glial scars play a beneficial role by containing the spread of inflammatory responses and providing structural support to the infarcted area. However, during the repair phase, these glial scars can impede neuronal reconnection and axonal extension. Additionally, reactive astrocytes often adopt a pro‐inflammatory profile, contributing to secondary brain injury [75]. The depletion of Tregs has been shown to increase the neurotoxic reactive astrocytes and deterioration of neurological function. Conversely, the adoptive transfer of Tregs in T‐cell‐deficient mice significantly alleviated astrogliosis [7]. Amphiregulin (AREG), an epidermal growth factor receptor (EGFR) ligand produced by Tregs, plays a critical role in modulating astrocyte activity by downregulating the expression of genes detrimental to neurons. Intraventricular administration of AREG in Treg‐depleted mice has been shown to alleviate astrogliosis and neuronal apoptosis, accompanied by improved neurological function following IS [7], suggesting Tregs exert their effects on reactive astrocytes, at least in part, through AREG (Figure 2).

Furthermore, IL‐10 has been shown to activate the phosphorylation of extracellular regulated protein kinases (ERK) and STAT3, and promote the proliferation and differentiation of neural stem cells (NSCs) in the subventricular zone (SVZ) [76, 77]. Since Tregs are a source of IL‐10, which increases during the chronic phase beginning 2 weeks after stroke [78], it suggests that Tregs may influence NSC activity and contribute to stroke recovery.

## 5 Therapeutic Strategies of Enhancing Treg Levels for IS

Based on the anti‐inflammatory effects, we propose that Tregs play a critical role in neuroprotection and functional recovery following IS. Although Treg levels initially decrease after stroke, they subsequently increase and remain elevated for an extended period, indicating a prolonged therapeutic window for Treg‐based treatments. This extended window suggests the potential for Treg cell therapy to enhance stroke rehabilitation. Several preclinical studies have explored the therapeutic potential of Treg cell therapy for IS (Table 1).

**TABLE 1:** Treg cell‐related therapies in IS.

Treatment	Animals	Model	Time point of treatment	Effects
Adoptive transfer of Tregs (iv)	Mice	tMCAO for 60 min	2, 4, 24 h after MCAO	Reduced brain infarct and prolonged improvement of neurological functions lasting out to 4 weeks [52]
	SD‐rats	tMCAO for 120 min		
Adoptive transfer of Tregs (iv)	Mice	tMCAO for 60 min	2 h after MCAO	Increased the number of Tregs in brain 7 days after tMCAO; improved in white matter integrity [79]
Intraperitoneal injection of IL‐2/IL‐2Ab complex	Mice	tMCAO for 60 min	Three days before MCAO or 2 h after MCAO	Increased the number of Tregs in the blood, spleen, and lymph nodes [80]
Intraperitoneal injection of IL‐2/IL‐2Ab complex	Mice	tMCAO for 60 min	For 3 consecutive days starting 6 h after tMCAO	Increased the number of Tregs in blood, spleen, and ischemic brain on 14 days; promoted white matter integrity in the late phase of stroke [79]
Astrocyte‐targeted gene delivery of IL‐2	Mice	PT and dMCAO		Increased the number of Tregs in brain without changing in peripheral immune cells; reduced brain infarct on 14 days after stroke [81]
Intraperitoneal injection of IL‐2/IL‐2Ab complex	Mice	tMCAO for 60 min	Starting 6 h after stroke, and repeated on days 1, 2, 3, 10, 20, and 30 after tMCAO	Increased the number of Tregs in blood, spleen, and ischemic brain; improved white matter integrity; improved sensorimotor functions [79]
Intraperitoneal injection of IL‐33	Mice	tMCAO for 60 min	Immediately after tMCAO and repeated once a day for 14 days	Increased the number of Tregs in spleen and brain; increased anti‐inflammatory factors such as IL‐10, TGF‐β, and IL‐35 [82]
Intracerebroventricular of IL‐33	Mice	tMCAO for 30 min	24, 48, and 72 h after reperfusion	Increased the number of Tregs in brain; increased anti‐inflammatory factors, AREG, and EGFR [83]
Intraperitoneal injection of IL‐33	Mice	tMCAO for 30 min	30 min before, and 30 min after tMCAO	Increased Tregs in the spleen; Increased IL‐4, IL‐10, and TGF‐β; reduced infarct volume; and improved neurological function [84]
Intraperitoneal injection of IL‐33	Mice	tMCAO for 60 min	24 h before and immediately after tMCAO	Increased Tregs in brain; reduced infarct volume; increased incidence of post‐stroke bacterial infection of the lungs [85]
Intraperitoneal injection of CD28SA	Mice	tMCAO for 30 min	Three days before tMCAO	Increased infarct volume; increasing thrombo‐inflammation [86]
Intraperitoneal injection of CD28SA	Mice	tMCAO for 60 min	3 and 6 h after tMCAO	Increased numbers of Tregs in spleen and brain; decreased infarct volume; and attenuated functional deficit [87]
Intraperitoneal injection of sRAGE	Mice	tMCAO for 60 min	15 min before MCAO plus 90 min after MCAO	Favored CD4^+^ T cells metabolic reprogramming and polarization toward Tregs; increased the proportion of Treg/Th17 cells in blood and spleen; improved long‐term neurological function [88]
Intraperitoneal injection of fingolimod	Yang mice, old mice, and hyperlipidemic mice	dMCAO	2 h before dMCAO plus 24 and 48 h after dMCAO	Increased the number of Tregs in blood, secondary lymphoid organs, and brain [89]
Intraperitoneal injection of fingolimod	Mice	dMCAO	Once a day for 10 days post‐dMCAO	Increased Tregs frequency in the periphery and brain; improved neuro‐behavioral recovery; improved the suppressive function of Tregs [90]

Abbreviations: dMCAO, distal middle cerebral artery occlusion; PT, photothrombotic stroke; tMCAO, transient middle cerebral occlusion.

### 5.1 Adoptive Transfer Therapy

The number of endogenous infiltrating Tregs is insufficient to achieve effective therapeutic outcomes in IS. Administrating exogenous Tregs can address this shortage. Circulating Tregs are the most readily accessible source for in vitro expansion; however, their low abundance and the risk of contamination with other T‐cell subsets during expansion pose challenges to their use [91, 92]. Human umbilical cord blood (UCB) contains a higher proportion of Tregs compared to peripheral blood, simplifying purification after expansion, making UCB a commonly used alternative source for Tregs [93]. Additionally, discarded human thymuses tissue offers an excellent source of Tregs, potentially overcoming the limitations associated with peripheral blood and UCB [94]. Another promising approach is Treg cell engineering, which involves the genetic modification of Tregs to introduce either a transgenic T‐cell receptor (TCR) or a chimeric antigen receptor (CAR). This enables Tregs to specifically recognize and respond to defined targets, enhancing their therapeutic potential [95].

Studies have demonstrated that systemic adoptive transfer of Tregs during the early phases (2, 6, and 24 h) after MCAO can reduce infarct size, mitigate brain damage, and improve neurological outcomes [52]. However, the potential benefits of adoptive Treg transfer during the rehabilitation phase after IS remain largely unexplored and warrant further investigation.

### 5.2 Stimulation In Vivo

In addition to increasing Treg numbers through exogenous methods, specific agents can stimulate endogenous Treg proliferation. IL‐2 has been shown to elicit robust Treg responses in vivo [96]. Intraperitoneal administration of IL‐2/IL‐2R antibody (IL‐2/IL‐2Ab) complex, either 3 days before MCAO or 2 h after MCAO, selectively increased Treg numbers in the blood, spleen, and lymph nodes. This approach also reduced infarct volume, suppressed neuroinflammation, and improved limb motor function [80]. A recent study demonstrated that systemic administration of the IL‐2/IL‐2Ab complex at multiple time points (6 h, 1 day, 2 days, 3 days, 10 days, 20 days, and 30 days) after MCAO significantly increased Treg levels in the blood, spleen, and brain. This treatment also promoted white matter integrity during the late phase of stroke recovery [79]. These findings suggest that the IL‐2/IL‐2Ab complex is a promising stimulant for enhancing rehabilitation after IS. The primary challenge of using IL‐2 to stimulate Tregs lies in its unintended activation of other immune cells [97]. To address this, targeted strategies have been developed. Combining recombinant IL‐2 with the mTOR inhibitor rapamycin and anti‐CD3/CD28 beads can achieve selective Treg proliferation by suppressing the proliferation of non‐Treg T‐cell subsets [98, 99]. Specifically, driving IL‐2 expression in astrocytes has been shown to selectively increase the number of brain‐resident Tregs [81]. Additionally, engineered IL‐2 biologics designed to selectively bind Tregs can expand their population in vivo without activating other components of the immune system, offering a more precise therapeutic approach [100].

IL‐33 is another common stimulant agent, as it facilitates Tregs expansion in ischemic brain tissue by activating Foxp3 expression [83]. Both intraperitoneal and intracerebroventricular administration of IL‐33 have been shown to increase the number of Tregs in the brain, reduce infarct volume, elevate levels of anti‐inflammatory cytokines such as IL‐10 and TGF‐β, and mitigate neurological deficits [82, 83, 84, 85]. However, IL‐33 administration is associated with an increased risk of post‐stroke bacterial lung infections [85]. An alternative approach involves the use of CD28 superagonistic monoclonal antibodies (CD28SA), which activate T cells independently of T‐cell receptor engagement. Intraperitoneal administration of CD28SA has been shown to increase Treg numbers in the spleen and brain, reduce infarct volume, and alleviate functional deficits [87]. However, while CD28 superagonist‐mediated boosting Treg levels can have therapeutic benefits, it has also been shown to worsen ischemic neurodegeneration and thrombo‐inflammation during the acute phase of IS [86]. The ischemic neurodegeneration might be attributed to the expansion of other immune cells triggered by CD28SA. The exacerbation of thrombo‐inflammation occurs because a significant number of retained Tregs in cerebral blood vessels can interact with vascular endothelial cells, leading to an increased risk of thrombosis [50]. Hence, the determination of a safe dosage for Tregs is a critical factor for their clinical application.

In addition, some new agents have also been found to stimulate endogenous Tregs. Administering soluble advanced glycation end‐products receptor (sRAGE) following MCAO promotes metabolic reprogramming of CD4^+^ T cells and their polarization toward Tregs. This treatment increases the Treg/Th17 cell ratio in the blood and spleen, ultimately improving long‐term neurological function [88]. Fingolimod has been shown to enhance both the number and functionality of Tregs in the blood, secondary lymphoid organs, and brain 7 days after permanent MCAO (pMCAO). By improving the immunosuppressive capabilities of Tregs, Fingolimod exerts a neuroprotective effect, mitigating ischemic injury [89, 90].

The preclinical studies have revealed that most therapeutic strategies for enhancing Treg levels in IS are effective, although some strategies may show some adverse reactions. Thereby countermeasures are required to ensure safety. Meanwhile, further research on the optimal dosage is required.

## 6 Conclusions

In summary, Tregs exert highly protective effects following IS due to their immunosuppressive function. Given the critical role of Treg infiltration into the brain during the rehabilitation phase, optimizing their delivery to brain tissue is essential for effective therapies. The restorative function for the delayed administration of the subacute and chronic phases following stroke needs to be further elucidated. Although some reports have investigated the infiltration of Tregs into the brain, the precise mechanisms by which Tregs cross the BBB remain unclear. Strategies that enhance Treg infiltration, potentially through chemokine modulation or receptor targeting, may guide Tregs across the BBB and achieve therapeutic effects. Combining biomaterials with Tregs offers another potential solution for improving targeted delivery.

Several clinical trials of Treg therapy for neurological disorders have been implemented, such as for AD (NCT03865017, NCT05016427), amyotrophic lateral sclerosis (NCT04220190, NCT06169176), and Kiran Ballet Syndrome (NCT03773328). Currently, the application of Tregs in IS remains predominantly in the preclinical research stage. Before advancing to clinical trials, several critical issues must be resolved. One major concern is the potential for Tregs to transdifferentiate into pro‐inflammatory Th17 cells in inflammatory environments, such as in the presence of IL‐6 stimulation [101]. To address this concern, TGF‐β stimulation prior to Treg therapy has been proposed as a preventive measure [102]. Another concern is that a systemic increase in Tregs may cause excessive immune suppression. Therefore, the timing and dose of Treg treatment has to be well controlled. The complexity of clinical scenarios also poses a significant hurdle. Most preclinical studies on Treg‐related therapies in IS utilize ischemia–reperfusion models, whereas many clinical cases involve cerebral ischemia without reperfusion. This distinction raises the question of whether Tregs function differently in nonreperfusion ischemia compared to reperfusion models. Further research is necessary to clarify the role and efficacy of Tregs under these differing conditions. Age and other high‐risk factors for cerebrovascular diseases—which are common in clinical practice—should be incorporated into animal models to better replicate patient conditions and establish more precise therapy parameters. Furthermore, the optimal treatment time window for Tregs intervention in stroke rehabilitation needs to be clarified. And the integration of Tregs with existing therapies and rehabilitation methods for IS requires further investigation to determine whether such combinations could produce synergistic effects. Finally, for Treg adoptive transfer therapy to become clinically viable, the source of Tregs must be standardized, and production must be scaled up to meet the therapeutic demands while ensuring safety, efficacy, and reproducibility.

## Conflicts of Interest

The authors declare no conflicts of interest.

## Data Availability

The data that support the findings of this study are available from the corresponding author upon reasonable request.

## References

[1] N. A. Hilkens , B. Casolla , T. W. Leung , and F. E. de Leeuw , “Stroke,” Lancet 403, no. 10446 (2024): 2820–2836. 38759664 10.1016/S0140-6736(24)00642-1

[2] R. Mao , N. Zong , Y. Hu , Y. Chen , and Y. Xu , “Neuronal Death Mechanisms and Therapeutic Strategy in Ischemic Stroke,” Neuroscience Bulletin 38, no. 10 (2022): 1229–1247. 35513682 10.1007/s12264-022-00859-0 PMC9554175

[3] K. L. Lambertsen , B. Finsen , and B. H. Clausen , “Post‐Stroke Inflammation‐Target or Tool for Therapy?,” Acta Neuropathologica 137, no. 5 (2019): 693–714. 30483945 10.1007/s00401-018-1930-z PMC6482288

[4] C. Iadecola , M. S. Buckwalter , and J. Anrather , “Immune Responses to Stroke: Mechanisms, Modulation, and Therapeutic Potential,” Journal of Clinical Investigation 130, no. 6 (2020): 2777–2788. 32391806 10.1172/JCI135530 PMC7260029

[5] Y. Liu , J. Dong , Z. Zhang , Y. Liu , and Y. Wang , “Regulatory T Cells: A Suppressor Arm in Post‐Stroke Immune Homeostasis,” Neurobiology of Disease 189 (2023): 106350. 37952680 10.1016/j.nbd.2023.106350

[6] M. Wang , A. W. Thomson , F. Yu , R. Hazra , A. Junagade , and X. Hu , “Regulatory T Lymphocytes as a Therapy for Ischemic Stroke,” Seminars in Immunopathology 45, no. 3 (2023): 329–346. 36469056 10.1007/s00281-022-00975-z PMC10239790

[7] M. Ito , K. Komai , S. Mise‐Omata , et al., “Brain Regulatory T Cells Suppress Astrogliosis and Potentiate Neurological Recovery,” Nature 565, no. 7738 (2019): 246–250. 30602786 10.1038/s41586-018-0824-5

[8] L. Shi , Z. Sun , W. Su , et al., “Treg Cell‐Derived Osteopontin Promotes Microglia‐Mediated White Matter Repair After Ischemic Stroke,” Immunity 54, no. 7 (2021): 1527–1542. 34015256 10.1016/j.immuni.2021.04.022 PMC8282725

[9] F. Yu , T. Huang , Y. Ran , et al., “New Insights Into the Roles of Microglial Regulation in Brain Plasticity‐Dependent Stroke Recovery,” Frontiers in Cellular Neuroscience 15 (2021): 727899. 34421544 10.3389/fncel.2021.727899 PMC8374071

[10] X. Song , R. Chen , J. Li , et al., “Fragile Treg Cells: Traitors in Immune Homeostasis?,” Pharmacological Research 206 (2024): 107297. 38977207 10.1016/j.phrs.2024.107297

[11] K. J. Wu , Q. F. Qian , J. R. Zhou , et al., “Regulatory T Cells (Tregs) in Liver Fibrosis,” Cell Death Discovery 9, no. 1 (2023): 53. 36759593 10.1038/s41420-023-01347-8 PMC9911787

[12] J. L. Trujillo‐Ochoa , M. Kazemian , and B. Afzali , “The Role of Transcription Factors in Shaping Regulatory T Cell Identity,” Nature Reviews Immunology 23, no. 12 (2023): 842–856. 10.1038/s41577-023-00893-7 PMC10893967 37336954

[13] C. S. Hsieh , Y. Liang , A. J. Tyznik , S. G. Self , D. Liggitt , and A. Y. Rudensky , “Recognition of the Peripheral Self by Naturally Arising CD25+ CD4+ T Cell Receptors,” Immunity 21, no. 2 (2004): 267–277. 15308106 10.1016/j.immuni.2004.07.009

[14] A. M. Thornton , P. E. Korty , D. Q. Tran , et al., “Expression of Helios, an Ikaros Transcription Factor Family Member, Differentiates Thymic‐Derived From Peripherally Induced Foxp3+ T Regulatory Cells,” Journal of Immunology 184, no. 7 (2010): 3433–3441. 10.4049/jimmunol.0904028 PMC3725574 20181882

[15] J. M. Weiss , A. M. Bilate , M. Gobert , et al., “Neuropilin 1 Is Expressed on Thymus‐Derived Natural Regulatory T Cells, but Not Mucosa‐Generated Induced Foxp3+ T Reg Cells,” Journal of Experimental Medicine 209, no. 10 (2012): 1723–1742. 22966001 10.1084/jem.20120914 PMC3457733

[16] R. Opstelten , S. de Kivit , M. C. Slot , et al., “GPA33: A Marker to Identify Stable Human Regulatory T Cells,” Journal of Immunology 204, no. 12 (2020): 3139–3148. 10.4049/jimmunol.1901250 32366581

[17] M. Kanamori , H. Nakatsukasa , M. Okada , Q. Lu , and A. Yoshimura , “Induced Regulatory T Cells: Their Development, Stability, and Applications,” Trends in Immunology 37, no. 11 (2016): 803–811. 27623114 10.1016/j.it.2016.08.012

[18] M. Panduro , C. Benoist , and D. Mathis , “Tissue Tregs,” Annual Review of Immunology 34 (2016): 609–633. 10.1146/annurev-immunol-032712-095948 PMC4942112 27168246

[19] Y. Li , Y. Lu , S. H. Lin , et al., “Insulin Signaling Establishes a Developmental Trajectory of Adipose Regulatory T Cells,” Nature Immunology 22, no. 9 (2021): 1175–1185. 34429546 10.1038/s41590-021-01010-3

[20] N. Ali , B. Zirak , R. S. Rodriguez , et al., “Regulatory T Cells in Skin Facilitate Epithelial Stem Cell Differentiation,” Cell 169, no. 6 (2017): 1119–1129. 28552347 10.1016/j.cell.2017.05.002 PMC5504703

[21] R. Sakai , K. Komai , M. Iizuka‐Koga , A. Yoshimura , and M. Ito , “Regulatory T Cells: Pathophysiological Roles and Clinical Applications,” Keio Journal of Medicine 69, no. 1 (2020): 1–15. 31353330 10.2302/kjm.2019-0003-OA

[22] M. Ito , K. Komai , T. Nakamura , T. Srirat , and A. Yoshimura , “Tissue Regulatory T Cells and Neural Repair,” International Immunology 31, no. 6 (2019): 361–369. 30893423 10.1093/intimm/dxz031

[23] R. D. Michalek , V. A. Gerriets , S. R. Jacobs , et al., “Cutting Edge: Distinct Glycolytic and Lipid Oxidative Metabolic Programs Are Essential for Effector and Regulatory CD4+ T Cell Subsets,” Journal of Immunology 186, no. 6 (2011): 3299–3303. 10.4049/jimmunol.1003613 PMC3198034 21317389

[24] J. Geng , R. Chen , F. F. Yang , et al., “CD98‐Induced CD147 Signaling Stabilizes the Foxp3 Protein to Maintain Tissue Homeostasis,” Cellular & Molecular Immunology 18, no. 12 (2021): 2618–2631. 34759371 10.1038/s41423-021-00785-7 PMC8632965

[25] H. Wang , Z. Wang , Q. Wu , Y. Yuan , W. Cao , and X. Zhang , “Regulatory T Cells in Ischemic Stroke,” CNS Neuroscience & Therapeutics 27, no. 6 (2021): 643–651. 33470530 10.1111/cns.13611 PMC8111493

[26] K. E. Olson , R. L. Mosley , and H. E. Gendelman , “The Potential for Treg‐Enhancing Therapies in Nervous System Pathologies,” Clinical and Experimental Immunology 211, no. 2 (2023): 108–121. 36041453 10.1093/cei/uxac084 PMC10019130

[27] R. Xu , N. A. Ebraheim , Y. Ou , and R. A. Yeasting , “Anatomic Considerations of Pedicle Screw Placement in the Thoracic Spine. Roy‐Camille Technique Versus Open‐Lamina Technique,” Spine 23, no. 9 (1998): 1065–1068. 9589548 10.1097/00007632-199805010-00021

[28] L. J. Chi , H. B. Wang , Y. Zhang , and W. Z. Wang , “Abnormality of Circulating CD4(+)CD25(+) Regulatory T Cell in Patients With Guillain‐Barre Syndrome,” Journal of Neuroimmunology 192, no. 1–2 (2007): 206–214. 17997492 10.1016/j.jneuroim.2007.09.034

[29] L. Sanvito , A. Makowska , N. Gregson , R. Nemni , and R. A. Hughes , “Circulating Subsets and CD4(+)CD25(+) Regulatory T Cell Function in Chronic Inflammatory Demyelinating Polyradiculoneuropathy,” Autoimmunity 42, no. 8 (2009): 667–677. 19886739 10.3109/08916930903140907

[30] F. He and R. Balling , “The Role of Regulatory T Cells in Neurodegenerative Diseases,” Wiley Interdisciplinary Reviews. Systems Biology and Medicine 5, no. 2 (2013): 153–180. 22899644 10.1002/wsbm.1187

[31] J. A. Saunders , K. A. Estes , L. M. Kosloski , et al., “CD4+ Regulatory and Effector/Memory T Cell Subsets Profile Motor Dysfunction in Parkinson's Disease,” Journal of Neuroimmune Pharmacology 7, no. 4 (2012): 927–938. 23054369 10.1007/s11481-012-9402-z PMC3515774

[32] C. Dansokho , D. Ait Ahmed , S. Aid , et al., “Regulatory T Cells Delay Disease Progression in Alzheimer‐Like Pathology,” Brain 139, no. Pt 4 (2016): 1237–1251. 26912648 10.1093/brain/awv408

[33] L. Mao , P. Li , W. Zhu , et al., “Regulatory T Cells Ameliorate Tissue Plasminogen Activator‐Induced Brain Haemorrhage After Stroke,” Brain 140, no. 7 (2017): 1914–1931. 28535201 10.1093/brain/awx111 PMC6059175

[34] X. Urra , A. Cervera , N. Villamor , A. M. Planas , and A. Chamorro , “Harms and Benefits of Lymphocyte Subpopulations in Patients With Acute Stroke,” Neuroscience 158, no. 3 (2009): 1174–1183. 18619524 10.1016/j.neuroscience.2008.06.014

[35] M. Santamaria‐Cadavid , E. Rodriguez‐Castro , M. Rodriguez‐Yanez , et al., “Regulatory T Cells Participate in the Recovery of Ischemic Stroke Patients,” BMC Neurology 20, no. 1 (2020): 68. 32111174 10.1186/s12883-020-01648-w PMC7048127

[36] K. Prass , C. Meisel , C. Hoflich , et al., “Stroke‐Induced Immunodeficiency Promotes Spontaneous Bacterial Infections and Is Mediated by Sympathetic Activation Reversal by Poststroke T Helper Cell Type 1‐Like Immunostimulation,” Journal of Experimental Medicine 198, no. 5 (2003): 725–736. 12939340 10.1084/jem.20021098 PMC2194193

[37] J. Yan , J. M. Greer , K. Etherington , et al., “Immune Activation in the Peripheral Blood of Patients With Acute Ischemic Stroke,” Journal of Neuroimmunology 206, no. 1–2 (2009): 112–117. 19058859 10.1016/j.jneuroim.2008.11.001

[38] J. Yan , S. J. Read , R. D. Henderson , et al., “Frequency and Function of Regulatory T Cells After Ischaemic Stroke in Humans,” Journal of Neuroimmunology 243, no. 1–2 (2012): 89–94. 22261543 10.1016/j.jneuroim.2011.12.019

[39] S. Li , Y. Huang , Y. Liu , et al., “Change and Predictive Ability of Circulating Immunoregulatory Lymphocytes in Long‐Term Outcomes of Acute Ischemic Stroke,” Journal of Cerebral Blood Flow and Metabolism 41, no. 9 (2021): 2280–2294. 33641517 10.1177/0271678X21995694 PMC8393304

[40] L. Gong , X. Zheng , W. Zhang , et al., “CD4(+)CD25(+) Regulatory T Cells in Intracranial Thrombi Are Inversely Correlated With Hemorrhagic Transformation After Thrombectomy: A Clinical‐Immunohistochemical Analysis of Acute Ischemic Stroke,” Oxidative Medicine and Cellular Longevity 2021 (2021): 3143248. 34055193 10.1155/2021/3143248 PMC8149217

[41] M. Lukasik , M. Telec , R. Kazmierski , et al., “Temporal Changes in Regulatory T Cell Subsets Defined by the Transcription Factor Helios in Stroke and Their Potential Role in Stroke‐Associated Infection: A Prospective Case‐Control Study,” Journal of Neuroinflammation 20, no. 1 (2023): 275. 37996909 10.1186/s12974-023-02957-w PMC10666369

[42] G. Deng , Y. Tang , J. Xiao , et al., “Naive‐Memory Regulatory T Cells Ratio Is a Prognostic Biomarker for Patients With Acute Ischemic Stroke,” Frontiers in Aging Neuroscience 15 (2023): 1072980. 36909948 10.3389/fnagi.2023.1072980 PMC9995800

[43] H. Offner , S. Subramanian , S. M. Parker , et al., “Splenic Atrophy in Experimental Stroke Is Accompanied by Increased Regulatory T Cells and Circulating Macrophages,” Journal of Immunology 176, no. 11 (2006): 6523–6531. 10.4049/jimmunol.176.11.6523 16709809

[44] T. Stubbe , F. Ebner , D. Richter , et al., “Regulatory T Cells Accumulate and Proliferate in the Ischemic Hemisphere for up to 30 Days After MCAO,” Journal of Cerebral Blood Flow and Metabolism 33, no. 1 (2013): 37–47. 22968321 10.1038/jcbfm.2012.128 PMC3597367

[45] M. Gelderblom , F. Leypoldt , K. Steinbach , et al., “Temporal and Spatial Dynamics of Cerebral Immune Cell Accumulation in Stroke,” Stroke 40, no. 5 (2009): 1849–1857. 19265055 10.1161/STROKEAHA.108.534503

[46] A. Liesz , E. Suri‐Payer , C. Veltkamp , et al., “Regulatory T Cells Are Key Cerebroprotective Immunomodulators in Acute Experimental Stroke,” Nature Medicine 15, no. 2 (2009): 192–199. 10.1038/nm.1927 19169263

[47] P. Li , L. Wang , Y. Zhou , et al., “C‐C Chemokine Receptor Type 5 (CCR5)‐mediated Docking of Transferred Tregs Protects Against Early Blood‐Brain Barrier Disruption After Stroke,” Journal of the American Heart Association 6, no. 8 (2017): 6387. 10.1161/JAHA.117.006387 PMC5586468 28768648

[48] S. Lee , J. Kim , J. S. You , Y. M. Hyun , J. Y. Kim , and J. E. Lee , “Ischemic Stroke Outcome After Promoting CD4+CD25+ Treg Cell Migration Through CCR4 Overexpression in a tMCAO Animal Model,” Scientific Reports 14, no. 1 (2024): 10201. 38702399 10.1038/s41598-024-60358-2 PMC11068779

[49] A. Liesz , W. Zhou , S. Y. Na , et al., “Boosting Regulatory T Cells Limits Neuroinflammation in Permanent Cortical Stroke,” Journal of Neuroscience 33, no. 44 (2013): 17350–17362. 24174668 10.1523/JNEUROSCI.4901-12.2013 PMC6618366

[50] C. Kleinschnitz , P. Kraft , A. Dreykluft , et al., “Regulatory T Cells Are Strong Promoters of Acute Ischemic Stroke in Mice by Inducing Dysfunction of the Cerebral Microvasculature,” Blood 121, no. 4 (2013): 679–691. 23160472 10.1182/blood-2012-04-426734 PMC3790947

[51] X. Ren , K. Akiyoshi , A. A. Vandenbark , P. D. Hurn , and H. Offner , “CD4+FoxP3+ Regulatory T‐Cells in Cerebral Ischemic Stroke,” Metabolic Brain Disease 26, no. 1 (2011): 87–90. 21082336 10.1007/s11011-010-9226-6 PMC3070853

[52] P. Li , Y. Gan , B. L. Sun , et al., “Adoptive Regulatory T‐Cell Therapy Protects Against Cerebral Ischemia,” Annals of Neurology 74, no. 3 (2013): 458–471. 23674483 10.1002/ana.23815 PMC3748165

[53] J. Wang , L. Xie , C. Yang , et al., “Activated Regulatory T Cell Regulates Neural Stem Cell Proliferation in the Subventricular Zone of Normal and Ischemic Mouse Brain Through Interleukin 10,” Frontiers in Cellular Neuroscience 9 (2015): 361. 26441532 10.3389/fncel.2015.00361 PMC4568339

[54] A. M. Gori , B. Giusti , B. Piccardi , et al., “Inflammatory and Metalloproteinases Profiles Predict Three‐Month Poor Outcomes in Ischemic Stroke Treated With Thrombolysis,” Journal of Cerebral Blood Flow and Metabolism 37, no. 9 (2017): 3253–3261. 28266892 10.1177/0271678X17695572 PMC5584701

[55] P. Li , L. Mao , X. Liu , et al., “Essential Role of Program Death 1‐Ligand 1 in Regulatory T‐Cell‐Afforded Protection Against Blood‐Brain Barrier Damage After Stroke,” Stroke 45, no. 3 (2014): 857–864. 24496394 10.1161/STROKEAHA.113.004100 PMC3939692

[56] P. Li , L. Mao , G. Zhou , et al., “Adoptive Regulatory T‐Cell Therapy Preserves Systemic Immune Homeostasis After Cerebral Ischemia,” Stroke 44, no. 12 (2013): 3509–3515. 24092548 10.1161/STROKEAHA.113.002637 PMC3895539

[57] B. Pu , H. Zhu , L. Wei , et al., “The Involvement of Immune Cells Between Ischemic Stroke and Gut Microbiota,” Translational Stroke Research 15, no. 3 (2024): 498–517. 37140808 10.1007/s12975-023-01151-7

[58] W. Zhang , R. Tang , Y. Yin , J. Chen , L. Yao , and B. Liu , “Microbiome Signatures in Ischemic Stroke: A Systematic Review,” Heliyon 10, no. 1 (2024): e23743. 38192800 10.1016/j.heliyon.2023.e23743 PMC10772200

[59] C. Benakis , D. Brea , S. Caballero , et al., “Commensal Microbiota Affects Ischemic Stroke Outcome by Regulating Intestinal Gammadelta T Cells,” Nature Medicine 22, no. 5 (2016): 516–523. 10.1038/nm.4068 PMC4860105 27019327

[60] R. Q. Zhou , “Relationship Between Serum GOT of Cyprinus Carpio and Biotic Index of Diatom in the Diagnosis of Water Quality,” Bulletin of Environmental Contamination and Toxicology 44, no. 6 (1990): 844–850. 2354259 10.1007/BF01702173

[61] C. Neumann , A. Scheffold , and S. Rutz , “Functions and Regulation of T Cell‐Derived Interleukin‐10,” Seminars in Immunology 44 (2019): 101344. 31727465 10.1016/j.smim.2019.101344

[62] S. Dolati , M. Ahmadi , M. Khalili , et al., “Peripheral Th17/Treg Imbalance in Elderly Patients With Ischemic Stroke,” Neurological Sciences 39, no. 4 (2018): 647–654. 29353353 10.1007/s10072-018-3250-4

[63] M. Wang , Y. Wang , J. He , et al., “Albumin Induces Neuroprotection Against Ischemic Stroke by Altering Toll‐Like Receptor 4 and Regulatory T Cells in Mice,” CNS & Neurological Disorders Drug Targets 12, no. 2 (2013): 220–227. 23394540 10.2174/18715273113129990058

[64] E. M. Shevach , “Mechanisms of foxp3+ T Regulatory Cell‐Mediated Suppression,” Immunity 30, no. 5 (2009): 636–645. 19464986 10.1016/j.immuni.2009.04.010

[65] A. Ricci and A. Liesz , “A Tale of Two Cells: Regulatory T Cell‐Microglia Cross‐Talk in the Ischemic Brain,” Science Translational Medicine 15, no. 721 (2023): eadj0052. 37939163 10.1126/scitranslmed.adj0052

[66] W. Lu , Y. Wang , and J. Wen , “The Roles of RhoA/ROCK/NF‐kappaB Pathway in Microglia Polarization Following Ischemic Stroke,” Journal of Neuroimmune Pharmacology 19, no. 1 (2024): 19. 38753217 10.1007/s11481-024-10118-w

[67] B. A. Resch , L. Kovacs , and J. G. Papp , “Prenatal Effect of Fenoterol (Partusisten) on the Heart in the Presence of Verapamil,” Zentralblatt für Gynäkologie 109, no. 23 (1987): 1446–1452. 3442154

[68] C. Qiao , Z. Liu , and S. Qie , “The Implications of Microglial Regulation in Neuroplasticity‐Dependent Stroke Recovery,” Biomolecules 13, no. 3 (2023): 571. 36979506 10.3390/biom13030571 PMC10046452

[69] X. Li , M. Yao , L. Li , et al., “Aloe‐Emodin Alleviates Cerebral Ischemia‐Reperfusion Injury by Regulating Microglial Polarization and Pyroptosis Through Inhibition of NLRP3 Inflammasome Activation,” Phytomedicine 129 (2024): 155578. 38621328 10.1016/j.phymed.2024.155578

[70] N. P. B. Au , T. Wu , G. Kumar , et al., “Low‐Dose Ionizing Radiation Promotes Motor Recovery and Brain Rewiring by Resolving Inflammatory Response After Brain Injury and Stroke,” Brain, Behavior, and Immunity 115 (2024): 43–63. 37774892 10.1016/j.bbi.2023.09.015

[71] C. Benakis , A. Simats , S. Tritschler , et al., “T Cells Modulate the Microglial Response to Brain Ischemia,” eLife 11 (2022): 11. 10.7554/eLife.82031 PMC9747154 36512388

[72] K. Zhou , Q. Zhong , Y. C. Wang , et al., “Regulatory T Cells Ameliorate Intracerebral Hemorrhage‐Induced Inflammatory Injury by Modulating Microglia/Macrophage Polarization Through the IL‐10/GSK3beta/PTEN Axis,” Journal of Cerebral Blood Flow and Metabolism 37, no. 3 (2017): 967–979. 27174997 10.1177/0271678X16648712 PMC5363473

[73] D. Brea , J. Agulla , M. Rodriguez‐Yanez , et al., “Regulatory T Cells Modulate Inflammation and Reduce Infarct Volume in Experimental Brain Ischaemia,” Journal of Cellular and Molecular Medicine 18, no. 8 (2014): 1571–1579. 24889329 10.1111/jcmm.12304 PMC4190903

[74] M. Pekny and M. Nilsson , “Astrocyte Activation and Reactive Gliosis,” Glia 50, no. 4 (2005): 427–434. 15846805 10.1002/glia.20207

[75] S. Xu , J. Lu , A. Shao , J. H. Zhang , and J. Zhang , “Glial Cells: Role of the Immune Response in Ischemic Stroke,” Frontiers in Immunology 11 (2020): 294. 32174916 10.3389/fimmu.2020.00294 PMC7055422

[76] F. J. Perez‐Asensio , U. Perpina , A. M. Planas , and E. Pozas , “Interleukin‐10 Regulates Progenitor Differentiation and Modulates Neurogenesis in Adult Brain,” Journal of Cell Science 126, no. Pt 18 (2013): 4208–4219. 23843621 10.1242/jcs.127803

[77] L. Pereira , M. Font‐Nieves , C. Van den Haute , V. Baekelandt , A. M. Planas , and E. Pozas , “IL‐10 Regulates Adult Neurogenesis by Modulating ERK and STAT3 Activity,” Frontiers in Cellular Neuroscience 9 (2015): 57. 25762897 10.3389/fncel.2015.00057 PMC4340210

[78] M. Kim , S. D. Kim , K. I. Kim , et al., “Dynamics of T Lymphocyte Between the Periphery and the Brain From the Acute to the Chronic Phase Following Ischemic Stroke in Mice,” Experimental Neurobiology 30, no. 2 (2021): 155–169. 33707347 10.5607/en20062 PMC8118758

[79] C. Yuan , L. Shi , Z. Sun , et al., “Regulatory T Cell Expansion Promotes White Matter Repair After Stroke,” Neurobiology of Disease 179 (2023): 106063. 36889482 10.1016/j.nbd.2023.106063

[80] H. Zhang , Y. Xia , Q. Ye , et al., “In Vivo Expansion of Regulatory T Cells With IL‐2/IL‐2 Antibody Complex Protects Against Transient Ischemic Stroke,” Journal of Neuroscience 38, no. 47 (2018): 10168–10179. 30291203 10.1523/JNEUROSCI.3411-17.2018 PMC6246882

[81] L. Yshii , E. Pasciuto , P. Bielefeld , et al., “Astrocyte‐Targeted Gene Delivery of Interleukin 2 Specifically Increases Brain‐Resident Regulatory T Cell Numbers and Protects Against Pathological Neuroinflammation,” Nature Immunology 23, no. 6 (2022): 878–891. 35618831 10.1038/s41590-022-01208-z PMC9174055

[82] X. Liu , R. Hu , L. Pei , et al., “Regulatory T Cell Is Critical for Interleukin‐33‐Mediated Neuroprotection Against Stroke,” Experimental Neurology 328 (2020): 113233. 32044328 10.1016/j.expneurol.2020.113233

[83] S. Guo and Y. Luo , “Brain Foxp3(+) Regulatory T Cells Can Be Expanded by Interleukin‐33 in Mouse Ischemic Stroke,” International Immunopharmacology 81 (2020): 106027. 31791672 10.1016/j.intimp.2019.106027

[84] W. Xiao , S. Guo , L. Chen , and Y. Luo , “The Role of Interleukin‐33 in the Modulation of Splenic T‐Cell Immune Responses After Experimental Ischemic Stroke,” Journal of Neuroimmunology 333 (2019): 576970. 31146104 10.1016/j.jneuroim.2019.576970

[85] S. R. Zhang , M. Piepke , H. X. Chu , et al., “IL‐33 Modulates Inflammatory Brain Injury but Exacerbates Systemic Immunosuppression Following Ischemic Stroke,” JCI Insight 3, no. 18 (2018): 560. 10.1172/jci.insight.121560 PMC6237219 30232272

[86] M. K. Schuhmann , P. Kraft , G. Stoll , et al., “CD28 Superagonist‐Mediated Boost of Regulatory T Cells Increases Thrombo‐Inflammation and Ischemic Neurodegeneration During the Acute Phase of Experimental Stroke,” Journal of Cerebral Blood Flow and Metabolism 35, no. 1 (2015): 6–10. 25315859 10.1038/jcbfm.2014.175 PMC4294400

[87] S. Y. Na , E. Mracsko , A. Liesz , T. Hunig , and R. Veltkamp , “Amplification of Regulatory T Cells Using a CD28 Superagonist Reduces Brain Damage After Ischemic Stroke in Mice,” Stroke 46, no. 1 (2015): 212–220. 25378432 10.1161/STROKEAHA.114.007756

[88] Y. Zhang , F. Li , C. Chen , et al., “RAGE‐Mediated T Cell Metabolic Reprogramming Shapes T Cell Inflammatory Response After Stroke,” Journal of Cerebral Blood Flow and Metabolism 42, no. 6 (2022): 952–965. 34910890 10.1177/0271678X211067133 PMC9125488

[89] K. Malone , A. C. Diaz Diaz , J. A. Shearer , A. C. Moore , and C. Waeber , “The Effect of Fingolimod on Regulatory T Cells in a Mouse Model of Brain Ischaemia,” Journal of Neuroinflammation 18, no. 1 (2021): 37. 33516262 10.1186/s12974-021-02083-5 PMC7847573

[90] K. Malone , J. A. Shearer , C. Waeber , and A. C. Moore , “The Impact of Fingolimod on Treg Function in Brain Ischaemia,” European Journal of Immunology 53, no. 9 (2023): e2350370. 37366289 10.1002/eji.202350370

[91] Y. Xia , W. Cai , A. W. Thomson , and X. Hu , “Regulatory T Cell Therapy for Ischemic Stroke: How Far From Clinical Translation?,” Translational Stroke Research 7, no. 5 (2016): 415–419. 27307291 10.1007/s12975-016-0476-4 PMC5179138

[92] C. Raffin , L. T. Vo , and J. A. Bluestone , “T(Reg) Cell‐Based Therapies: Challenges and Perspectives,” Nature Reviews Immunology 20, no. 3 (2020): 158–172. 10.1038/s41577-019-0232-6 PMC7814338 31811270

[93] R. Rikhtegar , M. Yousefi , S. Dolati , et al., “Stem Cell‐Based Cell Therapy for Neuroprotection in Stroke: A Review,” Journal of Cellular Biochemistry 120, no. 6 (2019): 8849–8862. 30506720 10.1002/jcb.28207

[94] I. E. Dijke , R. E. Hoeppli , T. Ellis , et al., “Discarded Human Thymus Is a Novel Source of Stable and Long‐Lived Therapeutic Regulatory T Cells,” American Journal of Transplantation 16, no. 1 (2016): 58–71. 26414799 10.1111/ajt.13456

[95] G. I. Ellis , N. C. Sheppard , and J. L. Riley , “Genetic Engineering of T Cells for Immunotherapy,” Nature Reviews Genetics 22, no. 7 (2021): 427–447. 10.1038/s41576-021-00329-9 PMC8217325 33603158

[96] D. A. Hurst and F. Sohrabji , “Interleukin‐2 Mediated Expansion of T‐Regulatory Cells as an Ischemic Stroke Therapy,” Stroke 55, no. 6 (2024): e159–e160. 38787931 10.1161/STROKEAHA.124.047357

[97] E. M. Shevach , “Application of IL‐2 Therapy to Target T Regulatory Cell Function,” Trends in Immunology 33, no. 12 (2012): 626–632. 22951308 10.1016/j.it.2012.07.007 PMC3505275

[98] L. Xie , F. Sun , J. Wang , et al., “mTOR Signaling Inhibition Modulates Macrophage/Microglia‐Mediated Neuroinflammation and Secondary Injury via Regulatory T Cells After Focal Ischemia,” Journal of Immunology 192, no. 12 (2014): 6009–6019. 10.4049/jimmunol.1303492 PMC4128178 24829408

[99] H. Fraser , N. Safinia , N. Grageda , et al., “A Rapamycin‐Based GMP‐Compatible Process for the Isolation and Expansion of Regulatory T Cells for Clinical Trials,” Molecular Therapy ‐ Methods & Clinical Development 8 (2018): 198–209. 29552576 10.1016/j.omtm.2018.01.006 PMC5850906

[100] R. Hernandez , J. Poder , K. M. LaPorte , and T. R. Malek , “Engineering IL‐2 for Immunotherapy of Autoimmunity and Cancer,” Nature Reviews. Immunology 22, no. 10 (2022): 614–628. 10.1038/s41577-022-00680-w 35217787

[101] N. Komatsu , K. Okamoto , S. Sawa , et al., “Pathogenic Conversion of Foxp3+ T Cells Into TH17 Cells in Autoimmune Arthritis,” Nature Medicine 20, no. 1 (2014): 62–68. 10.1038/nm.3432 24362934

[102] S. G. Zheng , J. Wang , and D. A. Horwitz , “Cutting Edge: Foxp3+CD4+CD25+ Regulatory T Cells Induced by IL‐2 and TGF‐Beta Are Resistant to Th17 Conversion by IL‐6,” Journal of Immunology 180, no. 11 (2008): 7112–7116. 10.4049/jimmunol.180.11.7112 18490709

